# From Tumor Macroenvironment to Tumor Microenvironment: The Prognostic Role of the Immune System in Oral and Lung Squamous Cell Carcinoma

**DOI:** 10.3390/cancers16152759

**Published:** 2024-08-03

**Authors:** Rosa Alessia Battista, Giacomo Maria Pini, Alex Finco, Filippo Corso, Andrea Galli, Gianluigi Arrigoni, Claudio Doglioni, Marcella Callea, Matteo Paccagnella, Luca Porcu, Federica Filipello, Marco Mazzola, Giorgia Foggetti, Vanesa Gregorc, Leone Giordano, Mario Bussi, Aurora Mirabile, Giulia Veronesi

**Affiliations:** 1 Faculty of Medicine and Surgery, Vita-Salute San Raffaele University, 20132 Milan, Italy; battista.rosaalessia@hsr.it (R.A.B.); finco.alex@hsr.it (A.F.); f.corso1@studenti.unisr.it (F.C.); galli1.andrea@hsr.it (A.G.); doglioni.claudio@hsr.it (C.D.); foggetti.giorgia@hsr.it (G.F.); giordano.leone@hsr.it (L.G.); bussi.mario@hsr.it (M.B.); veronesi.giulia@hsr.it (G.V.); 2 Department of Otolaryngology-Head and Neck Surgery, IRCCS San Raffaele Scientific Institute, 20132 Milan, Italy; 3 Department of Pathology, IRCCS San Raffaele Scientific Institute, 20132 Milan, Italy; pini.giacomo@hsr.it (G.M.P.); arrigoni.gianluigi@hsr.it (G.A.); callea.marcella@hsr.it (M.C.); ffilipello@aslcn2.it (F.F.); 4 Translational Oncology, ARCO Foundation, 12100 Cuneo, Italy; matteo.babeuf@gmail.com; 5 Cancer Research UK Cambridge Institute, Li Ka Shing Centre, University of Cambridge, Robinson Way, Cambridge CB2 0RE, UK; luca.porcu@cruk.cam.ac.uk; 6 Division of Pathology, Ospedale Michele e Pietro Ferrero, Verduno, 12060 Cuneo, Italy; 7 Department of Otolaryngology-Head and Neck Surgery, University of Verona, 37129 Verona, Italy; marco.mazzola@studenti.univr.it; 8 Division of Experimental Oncology, IRCCS San Raffaele Scientific Institute, 20132 Milan, Italy; 9 Department of Medical Oncology, IRCCS San Raffaele Scientific Institute, 20132 Milan, Italy; 10 Clinical Research and Innovation, Candiolo Cancer Institute, FPO-IRCCS, 10060 Candiolo, Italy; vanesa.gregorc@ircc.it; 11 Department of Thoracic Surgery, IRCCS San Raffaele Scientific Institute, 20132 Milan, Italy

**Keywords:** immune system, oral squamous cell carcinoma, lung squamous cell carcinoma, neutrophil-to-lymphocyte ratio, platelet-to-lymphocyte ratio, monocyte-to-lymphocyte ratio, tumor infiltrating lymphocytes, CD8+ lymphocytes, head and neck cancer, non-small cell lung cancer

## Abstract

**Simple Summary:**

This study explores the involvement of the immune system in oral and lung squamous cell carcinoma. Given the increasing recognition of the immune system’s significance in cancer, the study analyzes key components of both tumor macroenvironment and microenvironment. In detail, the focus is on systemic inflammation markers, such as Neutrophil-to-Lymphocyte Ratio (NLR), Platelet-to-Lymphocyte Ratio (PLR), and Monocyte-to-Lymphocyte Ratio (MLR). Additionally, this study investigates Tumor-Infiltrating Lymphocytes (TILs) and CD8+ cells in the tumor microenvironment. We aimed to better understand the impact of these prognostic factors on overall survival (OS) and disease-free survival (DSF) in order to enhance cancer risk stratification and guide therapeutic decisions.

**Abstract:**

Background: The interplay between cancer cells and the immune system is crucial in cancer progression and treatment. In this regard, the tumor immune microenvironment and macroenvironment, marked by systemic inflammation markers and TILs, could be considered key prognostic factors in tumors, including oral and lung squamous cell carcinoma. Methods: We conducted a retrospective clinical study on patients with Oral Squamous Cell Carcinoma (OSCC) and Lung Squamous Cell Carcinoma (LUSCC), examining stages, comorbidities, treatments, and outcomes. We evaluated the prognostic significance of pre-surgical systemic inflammation markers and tumor microenvironment composition. Results: Associations were found between systemic inflammation markers—NLR, MLR, and PLR—and tumor microenvironment factors, such as TILs and CD8+ cell prevalence—elevated inflammation markers correlated with advanced stages. Specifically, NLR was prognostic in OSCC, whereas PLR was prognostic in LUSCC. Using a cutoff value, we divided our tumor samples into two prognostic groups. Moreover, TILs levels >15% of tumor stroma correlated with prolonged overall survival in both OSCC and LUSCC, while increased CD8+ expression was linked to extended disease-free survival in LUSCC. Discussion: Systemic inflammation markers and TILs can be valuable prognostic factors of survival, highlighting the immune response’s role in OSCC and LUSCC. Despite limited clinical integration of the presented cohorts due to a lack of standardization, we concluded that analyzing tumor immune profiles may offer novel prognostic insights. Conclusions: Future integration into cancer classification could improve risk stratification and treatment guidance.

## 1. Introduction

The interplay between cancer cells and the immune system stands at the forefront of modern oncological studies. As underlined by Hiam-Galvez et al., the field of tumor immunology primarily concentrated on local immune responses within the *tumor microenvironment* (TME). However, immunity may be orchestrated across various tissues in the so-called *Tumor Macroenvironment* that comprehends not only the local TME but also blood and secondary lymphoid organs (bone marrow, lymph nodes, and spleen). From this perspective, both peripheral and tumor-infiltrating immune cells could be considered as potential subjects for in-depth study to gain a better understanding of their roles in combating tumors [1].

Amidst this complex interplay, systemic inflammation markers—namely, Neutrophil-to-Lymphocyte Ratio (NLR), Platelet-to-Lymphocyte Ratio (PLR), and Monocyte-to-Lymphocyte Ratio (MLR)—have gained recognition as potential, easily assessed, prognostic factors, expressing the inflammatory response to cancer [2,3,4,5,6,7]. Moreover, Tumor-Infiltrating Lymphocytes (TILs) and the density of different subpopulations of lymphocytes (such as CD3+, CD4+, and CD8+ T cells) within the tumor’s microenvironment have been traditionally associated with immune surveillance and cancer control. These elements of the immune system have been studied for their crucial potential role as prognostic biomarkers, offering insights into the adaptive immune response to tumor growth and progression. 

Of note, recent literature suggests a growing interest in integrating the prognostic significance of TNM classification, essentially based on *tumor-cell autonomous processes* (represented by dimension, invasiveness, and metastatic potential)*,* with the immunological, pathological features of various cancer types, which reflect *tumor-host relationship*, to enhance oncological risk stratification [8,9]. In fact, a global evaluation of host antitumor activity seems to be paramount in better-evaluating patients at high risk of disease progression and death. 

In particular, evaluation of TILs is possible and low-cost in Hematoxylin and Eosin (H&E)-stained sections. To further distinguish immune cell subtypes (CD4+ Th1, Th2, Treg, CD8+, NK, B cells), different Ab-labeled immunohistochemistry (IHC) is required. In colorectal cancer (CRC), Galon et al. first proposed in 2012 the adoption of a novel histological score, known as the *Immunoscore* [9], defined by the quantification of two specific lymphocyte populations, total CD3+ T cells and CD8+ effector T cells. They demonstrated the prognostic significance of this score, emphasizing the necessity for a more precise definition of prognostic stages in conjunction with the TNM classification. [8,10,11]. Accordingly, a consensus in 2017 assessed the role of TILs in various types of cancer, including breast cancer, melanoma, gastrointestinal tract carcinomas, non-small cell lung carcinoma (NSCLC), mesothelioma, endometrial and ovarian carcinomas, squamous cell carcinoma (SCC) of the head and neck (HN), genitourinary carcinomas, and primary brain tumors [12,13]. Despite the significant work conducted by this group, TILs evaluation is still not officially recognized in the 8th edition of the AJCC/UICC TNM staging system or the NCCN guidelines [14]. This may be due to the lack of a complete understanding of the role played by immune system cells within the TME as prognostic factors and the limited clinical translation of these findings. Moreover, the lack of standardization across different tumors in the evaluation of these prognostic immune biomarkers has contributed to their limited adoption in the oncological field. 

Thus, within this study, we aim to explore the dynamic roles of the immune system in SCC, particularly focusing on Oral Squamous Cell Carcinoma (OSCC) and Lung Squamous Cell Carcinoma (LUSCC) focusing on the established background of systemic inflammation markers and the immune cells implicated in the TME. Through this lens, we could review how these factors, namely the NLR, the PLR, and the MLR, may contribute to the prognosis of OSCC and LUSCC. We also assessed the potential role of TILs and CD8+ in these tumor settings.

## 2. Materials and Methods

### 2.1. Our Research Question: PICO Model

We conducted our research adhering to the *PICO model* guidelines [15]. The study focused on the Population of patients with Oral Squamous Cell Carcinoma (OSCC) and Lung Squamous Cell Carcinoma (LUSCC), including various stages of these cancers. The Interventions involved pre-surgical evaluations of systemic inflammation markers (NLR, MLR, PLR) and histological assessments of the tumor microenvironment, particularly focusing on Tumor-Infiltrating Lymphocytes (TILs) and CD8+ cell prevalence. For the Comparison, patients were divided into different prognostic groups based on cutoff values of these systemic inflammation markers and TIL levels, and these groups were compared for their outcomes. The main Outcomes measured were the prognostic significance of these interventions in predicting disease-free survival and overall survival.

### 2.2. Clinical Data Collection

Data from 104 consecutive patients who underwent surgery for OSCC at the Head and Neck Department of IRCCS San Raffaele Scientific Institute between 2008 and 2020 were gathered (median follow-up 90.1 months [95% confidence interval (CI): 62.5–119.4]). A total of 138 consecutive patients in the LUSCC group who underwent either diagnostic or therapeutic surgery at the Thoracic Surgery Department at IRCCS San Raffaele Scientific Institute between 2017 and 2023 were analyzed alongside (median follow-up 23.7 months [95% CI: 19.4–27.9]). Data encompassed clinical and pathological stages, patients’ comorbidities, risk factors, performance status, details of surgical treatment, assessment of surgical margins, and the administration of neoadjuvant or adjuvant chemotherapy, radiotherapy, or immunotherapy. Information on persistent disease, recurrent disease, or mortality was also compiled. 

### 2.3. Systemic Inflammatory Markers

We delved into the assessment of systemic inflammation through pre-surgical values of neutrophils, lymphocytes, monocytes, and platelets collected from the electronic health records of the participants. The following variables––Neutrophil-to-Lymphocyte Ratio (NLR), Monocyte-to-Lymphocyte Ratio (MLR), and Platelet-to-Lymphocyte Ratio (PLR)––were extracted and calculated by dividing the absolute neutrophil, monocyte, and platelet count by the absolute lymphocyte count obtained from complete blood count (CBC) results, respectively [2,3,4]. Cutoff values used to divide the cohorts of patients based on their risk of undergoing recurrence of disease or death were determined by Younden’s index.

### 2.4. Histopathological Evaluation

In a subgroup of patients randomly selected and representative of the entire sample (42/104 patients for OSCC and 32/138 patients for LUSCC), the tumor microenvironment was evaluated by quantifying TILs in Hematoxylin and Eosin (H&E)-stained sections and CD8-positive cells in immunohistochemistry (IHC). All the specimens were evaluated by two independent expert pathologists. Following the morphological review of the specimens, a representative section of the neoplasm was selected for each case. Tissue samples were fixed in buffered formalin and routinely processed to paraffin wax. Five-micrometer-thick sections were routinely stained with Hematoxylin and Eosin (H&E). Immunohistochemical (IHC) reactions were performed on additional 3-μm-thick sections using prediluted ready-to-use vials of the antibody [CD8, clone SP57, dilution RTU, Vendor: Ventana] with an automated immunostainer (BenchMark Ultra, Ventana Roche Diagnostics, Oro Valley, AZ, USA) and standardized protocols (Ventana OptiView DAB IHC Detection Kit).

TILs were evaluated employing the method established by the International Immuno-Oncology Biomarkers Working Group [12]. In summary, the entire slide was initially scanned at low magnification using a ×5 or ×10 objective lens, followed by a higher magnification with a ×20 objective lens. Stromal TILs were quantified as the percentage of the stromal area occupied by infiltrating lymphocytes. The average number of TILs was evaluated in multiple stromal regions, with scoring limited to mononuclear immune cells while excluding polymorphonuclear leukocytes. Additionally, areas of necrosis and stromal areas not directly adjacent to the tumor were excluded from the analysis. 

CD8+ lymphocytes were evaluated in IHC at the front of tumor invasion, defining four grades based on the number of cells positive on an average of five High Power Field (HPF), as described by Sakakura et al. [16]:

- Grade 1: <10 positive cells/HPF;
- Grade 2: 10–30 cells/HPF;
- Grade 3: 31–100 cells/HPF;
- Grade 4: >101 cells/HPF

### 2.5. Ethic Declaration

This study adhered to the principles outlined in the 1975 Declaration of Helsinki and received approval from the Institutional Ethics Committee (number 181/INT/2021). 

### 2.6. Statistical and Data Analysis

The distribution of continuous data was assessed using the Shapiro–Wilk test. Non-normally distributed variables were presented as median and interquartile range, while categorical variables were reported as absolute numbers and percentages. Mann–Whitney U test was employed for comparing continuous variables, and the Chi-square test was used for categorical variables. Overall survival (OS) and disease-free survival (DFS) were assessed through the unadjusted Kaplan–Meier method, and group survivals were compared using the log-rank test (Cox–Mantel test). Cox proportional hazards regression analysis was employed to identify significant predictors of endpoints. 

Variables with a univariate statistical significance of <0.05 were chosen for inclusion in the multivariable model. Multivariate analysis, utilizing stepwise forward selection, was ultimately conducted to analyze the association of baseline characteristics with study endpoints, expressed as hazard ratio (HR) with 95% confidence interval (CI) and *p* values. 

All statistical tests were two-sided, and *p* values <0.05 were deemed statistically significant. The statistical analyses were performed using SPSS software version 25.0.0 (SPSS Inc., Chicago, IL, USA) and GraphPad Prism software version 6 (GraphPad, Inc., San Diego, CA, USA).

## 3. Results

### 3.1. Clinical Data

The median age at the initiation of treatment for the entire OSCC cohort was 67 years (95% CI: 19–96 years). Gender distribution in the OSCC cohort was relatively balanced, with 51 males (49%) and 53 females (51%). The study uncovered a median overall survival (OS) of 96.0 months (95% CI: 50.3–141.6) and a median disease-free survival (DFS) of 26.5 months (95% CI: 9.7–43.3) among patients with OSCC. 

Regarding the LUSCC cohort, the median age at the start of treatment was 72 years for the entire cohort, ranging from 33 to 87. The male-to-female ratio was 81% to 19%. The analysis of OS revealed a median duration of 61.2 months, with a 95% CI ranging from 19.9 to 102.5 months. When exploring DFS, the median duration was found to be 31.7 months, with a 95% confidence interval spanning from 24.1 to 39.3 months. Full clinical data are reported in Supplementary Tables S1 and S2.

### 3.2. Evaluation of the Systemic Immune Response: Tumor Macroenvironment 

We investigated the role of systemic inflammation and immune host response in patients with OSCC and LUSCC through established hematological parameters proposed in the literature as potential markers of the inflammatory response to cancer: Neutrophil-to-Lymphocyte Ratio (NLR), Platelet-to-Lymphocyte Ratio (PLR), and Monocyte-to-Lymphocyte Ratio (MLR) [2,3,4].

**Figure 1:**
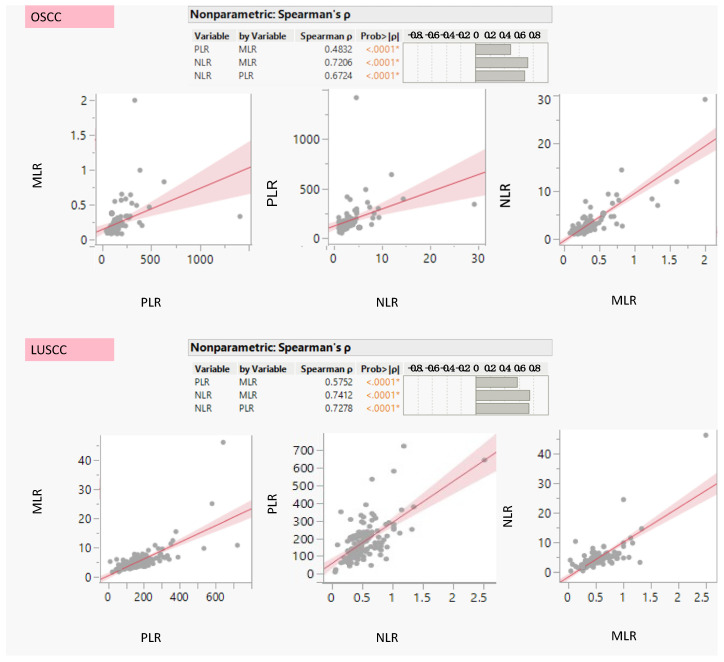
Spearman’s correlation between Systemic Inflammatory Markers Neutrophil-to-Lymphocyte Ratio (NLR), Monocyte-to-Lymphocyte Ratio (MRL), and Platelet-to-Lymphocyte Ratio (PLR) in Oral Squamous Cell Carcinoma (OSCC) and Lung Squamous Cell Carcinoma (LUSCC). (Each dot in the graph represents the Spearman correlation coefficient between a pair of systemic inflammatory markers (NLR, MLR, PLR) for either OSCC or LUSCC patients. Red lines show the line of best fit between two markers. Pink-shaded areas highlight the 95 % C.I. of the line of best fit between the systemic inflammatory markers; * stands for *p* value < 0.05)

By comparing these parameters, we observed interesting correlations between these parameters. In fact, the Spearman correlation analysis revealed significant relationships among NLR, MLR, and PLR in our dataset (Figure 1). In particular, we found a strong positive correlation between NLR and MLR in both OSCC and LUSCC, with a coefficient of 0.720 and 0.741, respectively, and a highly significant *p*-value (<0.001). This could indicate that an increase in NLR may be associated with a proportional increase in MLR. Similarly, a robust positive correlation was observed between NLR and PLR, with a correlation coefficient of 0.672 and 0.728 and a highly significant *p*-value of less than 0.001 in OSCC and LUSCC, respectively. This correlation could suggest that variations in NLR coincide with corresponding variations in PLR. Additionally, a significant positive correlation existed between MLR and PLR, though slightly less pronounced compared to the NLR-MLR and NLR-PLR associations. The correlation coefficient for MLR and PLR is 0.483 in OSCC and 0.575 in LUSCC, with a *p*-value less than 0.001, indicating that changes in MLR are positively associated with changes in PLR. These findings collectively imply a cohesive relationship between these immune-related ratios, signifying shared underlying mechanisms of these inflammatory markers. The results provide valuable insights into the interconnected dynamics of these ratios within the immune and inflammatory contexts in the studied population.

**Figure 2:**
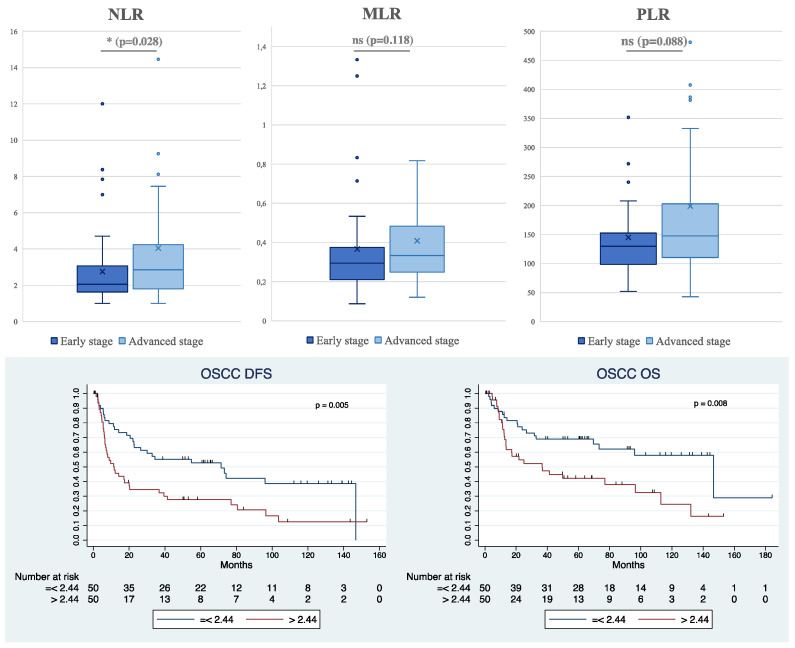
At the (**top**): Boxplots illustrating the distribution of NLR, MLR, and PLR values between early and advanced stages of patients with OSCC (“x” represents the mean values for each boxplot; external dots represents outliers; * highlights *p* value <0.05; ns = non-significant *p* value). At the (**bottom**): Kaplan–Meier survival curves depicting disease-free survival (DFS) and overall survival (OS) in OSCC based on NLR levels (<2.44 and >2.44), with corresponding *p*-values (DFS: *p* = 0.005, OS: *p* = 0.008).

**Figure 3:**
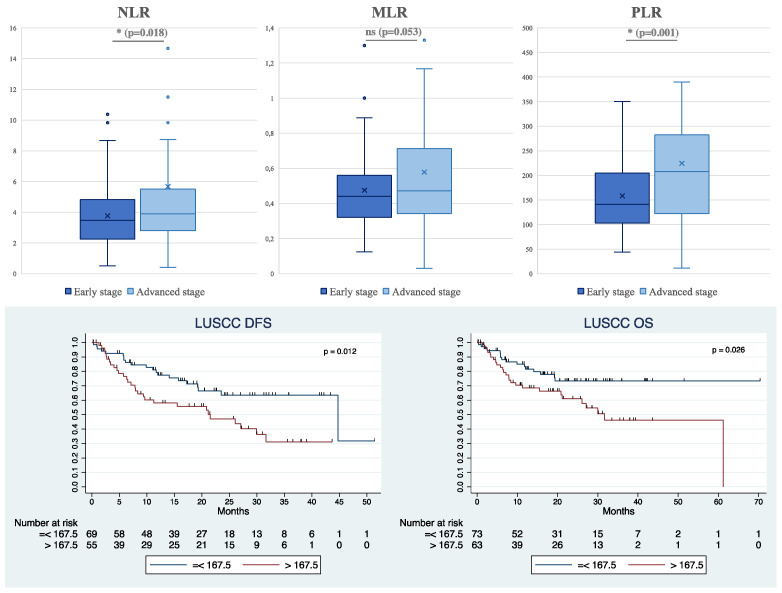
At the (**top**): Boxplots illustrating the distribution of NLR, MLR, and PLR values between early and advanced stages of Lung Squamous Cell Carcinoma (LUSCC) patients (“x” represents the mean values for each boxplot; external dots represents outliers; * highlights *p* value <0.05; ns = non-significant *p* value). At the (**bottom**): Kaplan–Meier survival curves depicting disease-free survival (DFS) and overall survival (OS) in LUSCC based on PLR levels (cutoff 167.5), with corresponding *p*-values (DFS: *p* = 0.012, OS: *p* = 0.026).

Furthermore, we observed that overall, all inflammatory markers were higher in patients with advanced-stage OSCC (Stages III–IV) or LUSCC (IIIb–IV) compared to those in early stages (I–II for OSCC and I–IIIa for LUSCC) (Figure 2 and Figure 3). However, in the OSCC group, only NLR exhibited a significant difference, with a mean of 2.76 ± 2.07 in the early stages compared to 4.04 ± 4.47 in the advanced stages (*p* = 0.028) (Figure 2). In the LUSCC population, we identified a statistically significant difference in both NLR and PLR parameters (Figure 3). Specifically, the mean values for early and advanced stages were 3.76 ± 1.95 and 5.67 ± 6.99, respectively (*p* = 0.018) for NLR, indicating a substantial variation. Similarly, for PLR, the means were 158.34 ± 70.73 and 224.22 ± 148.30 (*p* = 0.001), emphasizing a significant difference in PLR dynamics between early and advanced stages. It is noteworthy that, upon consideration of histological grading, no differences were observed in NLR, MLR, and PLR variables. This indicates that the immune-related ratios did not vary significantly based on the corresponding histological grading in our sample.

Subsequently, a cumulative analysis considering only the NLR values for every stage of the disease in OSCC revealed a significant association between NLR values and prognosis (Figure 2). Specifically, higher NLR values were associated with a worse prognosis. We found a cutoff value of NLR = 2.44. Accordingly, patients with NLR < 2.44 demonstrated a longer DFS of 71.4 [26.1–116.7] months compared to those with NLR above the cutoff value (11.7 [4.9–18.6] months) with a *p*-value of 0.005. A similar trend was observed in OS, with a median of 146.7 months [75.0–218.3] in patients with a lower NLR compared to 36.7 months [7.9–65.5] (*p*-value = 0.008).

In the context of LUSCC, the PLR serves as a significant parameter for predicting prognosis. In fact, putting a cutoff value of PLR = 167.5, a better prognosis was found in patients with lower PLR values in both DFS (*p* = 0.012) and OS (*p* = 0.026). For individuals with a PLR < 167.5, the median DFS is notably prolonged, measuring at 44.8 months [15.0–74.5]. Conversely, for those with a PLR exceeding 167.5, the median DFS significantly decreases to 21.5 months [8.6–34.5]. In summary, these results suggest a potential prognostic value of PLR in LUSCC. We further explored potential correlations between PDL1 expression in LUSCC and these ratios since they have also shown prognostic significance in progression-free survival in patients affected by NSCLC with PDL1 > 50%, treated with Pembrolizumab [17]. However, our analysis revealed the absence of any correlation between the expression of PDL1 and the immune-related parameters NLR, MLR, and PLR. 

Altogether, these findings contribute valuable insights for risk stratification and prognostic assessments in OSCC and LUSCC patients. Notably, these ratios showed statistical significance in a univariate analysis; however, when examined in a multivariate setting, we did not confirm the same significance. This may be related to a strong impact of staging classification on prognosis. 

### 3.3. Role of Tumor Microenvironment in Lung SCC and Oral SCC

We then focused our attention on the Tumor immune microenvironment, in particular TILs and CD8+ T cells. The Spearman correlation results reveal a statistically significant correlation (r = 0.441, *p* = 0.013) between Tumor-Infiltrating Lymphocytes and CD8 expression. This suggests a positive relationship between the presence of TILs and the expression of CD8. Being CD8+ T cells part of the TILs, this result is in line with what we have expected and helps interpreting the following results. 

### 3.4. Tumor-Infiltrating Lymphocytes as Potential Prognostic Indicators

**Figure 4:**
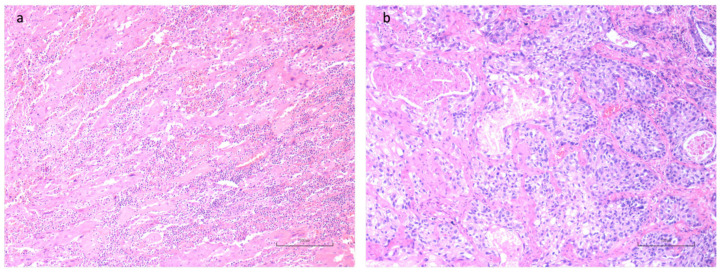
Representative histological sections for TILs evaluation in LUSCC: (**a**) High presence of TILs (60%) compared to (**b**) Low presence of TILs (5%), Hematoxylin and Eosin staining, 10×.

**Figure 5:**
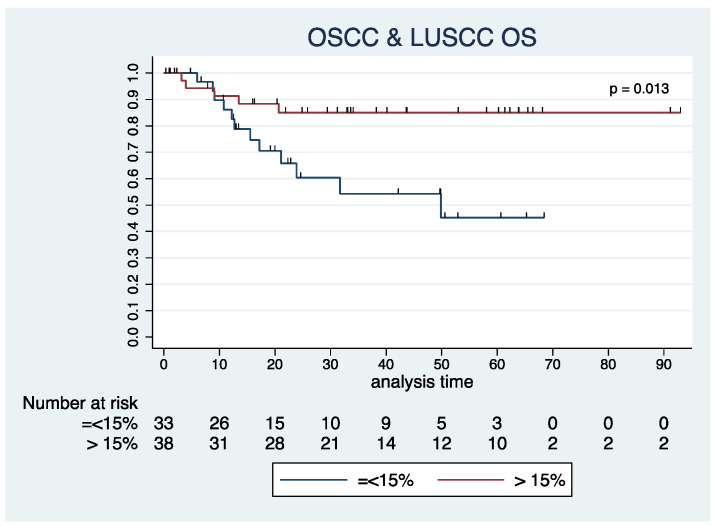
Kaplan–Meier curves illustrating overall survival (OS) in the combined Oral Squamous Cell Carcinoma (OSCC) and Lung Squamous Cell Carcinoma (LUSCC) cohort. The curves represent the subgroups of patients categorized based on Tumor-Infiltrating Lymphocytes (TILs) levels within the tumor stroma, specifically TILs ≤ 15% and TILs > 15%.

Importantly, we observed that the presence of TILs emerges as a significant predictor for OS, considering the two populations combined (Figure 4). In particular, the prognostic role of TILs in OS is highlighted by high significance (*p* = 0.013). For individuals with TILs constituting less than or equal to 15% of the tumor stroma, the median OS stands at 43.1 months, with a CI spanning from 32.7 to 53.5 months. In contrast, tumor patients with a TIL exceeding 15% experienced a substantially prolonged median OS of 80.4 months, with a CI ranging from 70.4 to 90.5 months (Figure 5).

Collectively, these results suggest a significant association between higher TIL levels and prolonged OS in the combined population of OSCC and LUSCC. 

The observed trend in the prognostic role of TILs in OS was not confirmed when assessing DFS. This lack of consistency was observed both in the OSCC and the LUSCC cohorts. In terms of DFS, TILs failed to demonstrate a significant association with survival outcomes in these populations. This discrepancy highlights the nuanced nature of immune responses and their impact on different aspects of cancer progression. 

### 3.5. CD8+ Cells

**Figure 6:**
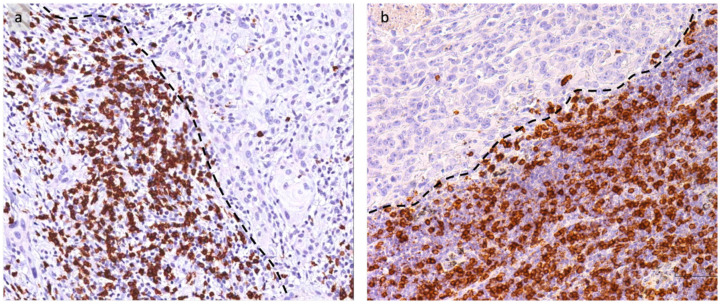
Representative histological sections for CD8+ lymphocytes evaluation at the invasive tumoral margin (dotted line) in OSCC (**a**) and LUSCC (**b**), CD8+ immunohistochemistry (IHC), 20×.

**Figure 7:**
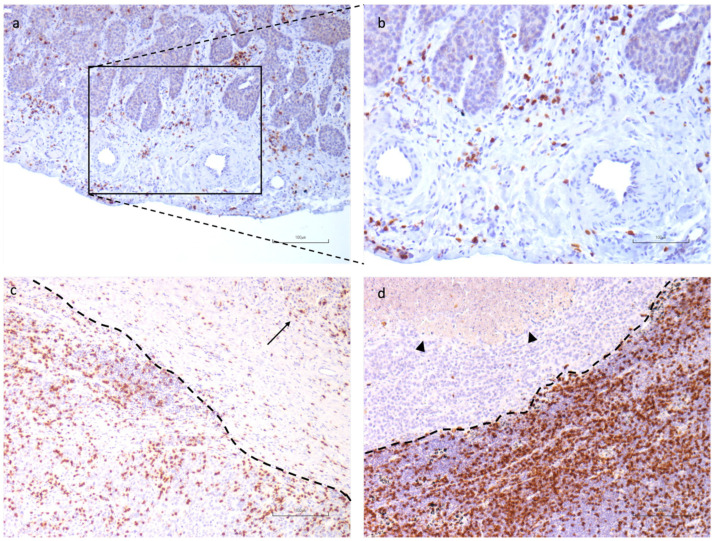
Representative histological sections for CD8+ lymphocytes evaluation at the invasive tumoral margin in LUSCC: CD8+ immunohistochemistry (IHC) (**a**) Grade 2 CD8 positivity, 10×; (**b**) same section, 20×, very few CD8+ lymphocytes at the tumor margin; (**c**) Grade 3 CD8 positivity at the invasive tumor margin (dotted line); arrow highlights nests of Lung Squamous Cell Carcinoma. Notably, mononuclear cells at the invasive tumoral margin consist mostly of CD8+ lymphocytes; (**d**) Grade 4 CD8+ cells at the tumor margin (dotted line), with an area of necrosis in the left superior corner (arrowheads).

We further evaluate CD8+ lymphocytes at the leading edge of tumor invasion (Figure 6) to categorize this cell population into four grades based on the number of positive cells observed on average across five High Power Fields (HPF), as shown in Figure 7 and reported in Section 2.

Notably, in LUSCC, the presence of CD8 positivity in the tumor immune microenvironment emerges as a significant prognostic factor for DFS. 

The median DFS for patients with low CD8+ expression (grade 1–2, less than 30 cells/HPF) is notably shorter, measuring at 2.8 months [CI 0.2–5.3 months]. In contrast, for individuals with CD8+ expression exceeding this cutoff (grade 3–4, >30 cells/HPF), the median DFS significantly extends to 40.6 months [CI 36.7–44.5 months]. The observed *p*-value, being <0.001, indicates a highly statistically significant difference in DFS between the two groups. This underscores the prognostic relevance of CD8 positivity in LSCC, with higher CD8 expression levels associated with a significantly prolonged DFS compared to lower expression levels.

It is noteworthy that the same trend was not replicated in the population with OSCC. The absence of a similar trend in the OSCC group may suggest either that the prognostic significance of CD8 positivity may vary across different types of squamous cell carcinomas or the possible limited sample in our OSCC study. This highlights the importance of considering tumor-specific factors and characteristics that contribute to the heterogeneous nature of cancer and underscores the need for distinct prognostic markers in different cancer types.

**Figure 8:**
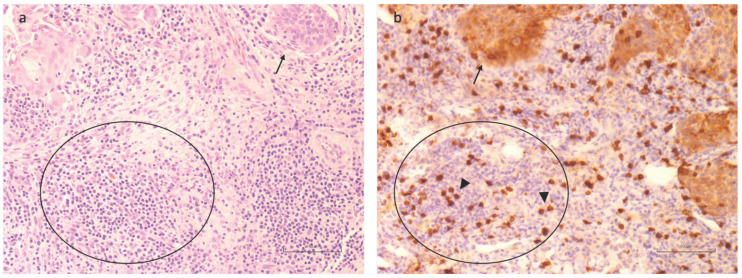
Representative histological sections for CD8+ lymphocytes evaluation at the invasive tumoral margin in LUSCC: (**a**) Hematoxylin and Eosin staining, 20×, (**b**) CD8+ immunohistochemistry (IHC), 20×. Arrows highlight nests of Lung Squamous Cell Carcinoma, while circles indicate one of the regions of the infiltrative tumoral margin rich in lymphocytes. Notably, consecutive sections of the same sample stained with an anti-CD8 ab reveal that these mononucleate cells are only in part CD8+ lymphocytes (arrowhead).

Finally, some descriptive observations highlighted a great variance of CD8 positivity in the context of TILs, meaning that the remaining uncharacterized populations may influence the TME. A particular observation was made upon the positivity of CD8+ expressed in the setting of TILs. This last parameter was not evaluated as a single variable since we focused our attention on the tumor-infiltrating margin for the evaluation of CD8+ lymphocytes. However, As a representative example, we show in Figure 8 the relatively low positivity of CD8 in the contest of TILs in a LUSCC sample. Such descriptive findings raise important considerations regarding the potential impact of various TIL subpopulations on the tumor environment and, consequently, on the prognosis and therapeutic response.

## 4. Discussion

Squamous cell carcinoma represents a pivotal challenge in oncology, with its intricate interactions within the tumor microenvironment and macroenvironment playing crucial roles in its progression and response to therapy. Traditionally, cancer research and treatments have focused on the tumor itself—its genetics, mutations, and direct environment. However, the evolving landscape of oncology now underscores a broader perspective, acknowledging the significant impact of the immune system, both locally within the tumor and systemically throughout the body.

Cancer induces a plethora of systemic changes, impacting various facets of the immune system. The disruption of hematopoiesis within the bone marrow leads to an increased proliferation of hematopoietic stem cells and granulocyte-monocyte progenitors (GMPs), resulting in a surge of immature immunosuppressive cells within both the bloodstream and the TME. Furthermore, the levels of peripheral blood dendritic cells (DCs), crucial for T-cell priming and differentiation, are notably reduced. T cells, vital for an effective immune response, also undergo significant changes in their subpopulations, alongside a functional perturbation marked by altered cytokine secretion and the expansion of suppressive regulatory T cells (Tregs), further facilitating tumor immune evasion.

These alterations can potentially transform peripheral blood cells into both prognostic factors and therapeutic targets. 

In the realm of systemic inflammation markers, the Neutrophil-to-Lymphocyte Ratio (NLR), Platelet-to-Lymphocyte Ratio (PLR), and Monocyte-to-Lymphocyte Ratio (MLR) stand out as significant prognostic factors. Their ease of measurement through routine blood tests makes them invaluable tools in assessing the systemic immune response to cancer. Elevated NLR, for instance, is associated with a general inflammatory state [18] and has been linked to worse prognoses in various cancers. The balance between neutrophils and lymphocytes—integral components of the innate and adaptive immune responses, respectively—offers insights into the body’s overall immune state and its capacity to combat tumor growth. This balance explains the prognostic significance of these markers, as corroborated in the literature. In fact, a systematic review updated to 2014, encompassing various solid tumors, such as pancreatic cancer, renal cell carcinoma, carcinoma of the colon and the rectum, gastroesophageal cancer, NSCLC, mesothelioma, cholangiocarcinoma, and hepatocellular carcinoma, underscored the adverse prognostic significance of the NLR in a cohort exceeding 40,000 oncological patients. In all oncologic categories according to tumor sites, stage, and histology, NLR greater than a median cutoff of 4 had a significant hazard ratio for OS, disease-specific survival (DSS), progression-free survival (PFS), and DFS [3,19]. Considering more specifically the district of interest of our study, a recent meta-analysis on Head and Neck Squamous Cell Carcinoma (HNSCC) was conducted by Mariani et al. in 2021, focusing on surgical patients who underwent or did not undergo adjuvant therapy, excluding cases of HPV+ cancers. They analyzed a total of 4597 and 2020 patients for OS and DFS, respectively, confirming in HNSCC the well-established trend of a worse prognosis in terms of OS (HR 1.56, *p* < 0.001) and DFS (HR 1.64, *p* < 0.0001) in patients with higher preoperative NLR values [20]. Additionally, in 2022, the group led by Takenaka investigated the role of NLR in predicting response to therapy and subsequent OS and PFS in patients affected by recurrent or metastatic HNSCC treated with Immune Checkpoint Inhibitors, discovering in a metanalysis that higher NLR was significantly associated with all endpoints (OS, HR 2.03; PFS, HR 2.15; response to therapy, OR 0.49; and disease control, OR 0.3) [21]. These findings were corroborated by Kang’s group in a comprehensive meta-analysis on prognostic biomarkers predicting response to Immune Checkpoint Inhibitors (ICIs) treatment in HNSCC [22]. Moreover, there are other biomarkers considered as surrogates for the immune system’s response to tumor burden, with a promising role in risk stratification. Specifically, the Platelet-to-Lymphocyte Ratio, which typically indicates an elevated platelet count (thrombophilia) and low lymphocyte count in blood samples (lymphopenia), may reflect an imbalance in the immune system response, impacting the adequate control of tumor cells. In 2018, Li et al. demonstrated a significant association of high PLR with poor OS and PFS in patients with advanced cancers, including NSCLC, nasopharyngeal cancer, renal cell carcinoma, pancreatic cancer, biliary tract cancer, liver cancer, gastric cancer, and CRC [4]. Similar studies conducted in NSCLC have affirmed the prognostic significance of NLR and PLR, even in these types of tumors [23]. In 2015, a meta-analysis made by Yin et al. on 14 previous studies demonstrated the significance of NLR in predicting prognosis for Lung cancer. The analysis encompassed both NSCLC and Small-cell Lung Cancer (SCLC) across both early and advanced stages [24]. In 2022, a meta-analysis published by Platini et al. examined 12 studies involving a total of 1719 patients affected by advanced NSCLC and treated with ICIs. The results consistently validated the prognostic value of high NLR and PLR in predicting poor outcomes in terms of OS and PFS [25]. 

In the present study, we confirmed the prognostic relevance of NLR and PLR in two of the most frequent SCCs: the oral (OSCC) and lung (LUSCC). Moreover, we identified a cutoff value that effectively discriminates against patients at a higher risk of disease recurrence or death, and our results are completely in line with previous literature. These findings highlight the crucial role of these easy-to-assess inflammatory preoperative biomarkers, which can potentially lead to tailored, more aggressive, and multimodal treatments for patients in high-risk groups.

Focusing on local immune response, TILs and different subpopulations in the TME are demonstrating a promising role in predicting prognosis in various oncological studies [12,13]. With his studies in 2012, Galon et al. were a pioneer in this field, highlighting the prognostic significance of TIL subpopulations assessed in the tumor core and at the invasive margin, proposing a novel histological score, known as the *Immunoscore* [10], which may implement the dimensional colorectal cancer (CRC) TNM. In fact, CD3+ counts were revealed to possess an independent prognostic value for DFS and OS in multivariate analysis, surpassing TNM classification in CRC [8,11]. Likewise, the International Immuno-Oncology Biomarker Working Group proposed in 2015 the standardization of TIL evaluation as a prognostic biomarker in breast cancer [26,27]. However, in this case, the TILs evaluation was recommended on simple H&E staining, exclusively in the stromal compartment, without the use of lymphocytic markers in IHC [28]. In 2017, a comprehensive review was conducted to assess the role of TILs in various types of cancer [12,13]. For melanoma, the inclusion of TIL information in pathological reports has become a routine practice, highlighting its prognostic significance and potential as a predictive marker for immunotherapy response. However, it has not yet been officially recognized in the 8th edition of the AJCC/UICC TNM staging system or the NCCN guidelines for melanoma treatment [14].

In the context of gastric cancer, Zhang et al. introduced a scoring system in 2019 inspired by Galon’s studies. This system was applied to TILs assessed on H&E slices, considering both the intensity and percentage of TILs in the CT and IM. Their findings revealed noteworthy prognostic implications, showing a significant association between TIL levels (high or low) and various clinicopathological factors, such as the dimension of the tumor, histological grading, involvement of regional lymph nodes, perineural invasion, tumor thrombus, pathological TNM (pTNM) stage, and World Health Organization (WHO) subtypes (*p* < 0.001). Furthermore, a high level of TILs demonstrated a positive predictive impact on overall survival (OS) in both Kaplan–Meier and multivariate analyses [29]. In 2015, the Donnem group showed how CD8+ TILs emerged as an independent prognostic factor in non-small cell lung cancer for all endpoints (OS, DFS, DSS with *p* < 0.001). In multivariate analysis, this variable demonstrated prognostic significance independent of pStage [30]. Additionally, they introduced the TNM-Immunoscore (TNM-I) for NSCLC, drawing on prior experience in colorectal cancer [8] and guidelines proposed for gastric cancers [29]. To simplify the evaluation, TILs were assessed in a unified compartment, considering the total expression in both the intraepithelial and stromal compartments. This approach holds the potential for easier use, especially in automated analyses, while maintaining the same prognostic impact [31]. Accordingly, in 2017, de Ruiter et al. conducted a meta-analysis on head and neck SCC (HNSCC), emphasizing the prognostic importance of CD3+ TILs in OS with a HR of 0.63 and DFS with a HR of 0.64. Additionally, CD8+ TILs were found to be prognostically significant in OS with a HR of 0.67 and in DFS with a HR of 0.50. This analysis encompassed various types of Head and Neck Squamous Cell Carcinoma (HNSCC), including those affecting the oral cavity, oropharynx, hypopharynx, and larynx [32]. In 2021, Borsetto et al. conducted a systematic review and meta-analysis among all anatomical sites of HNSCC, analyzing 28 selected studies. A high level of CD4+ and CD8+ TILs was associated with a reduced risk of death when considering various HN sites combined. However, while OPSCC and hypopharyngeal SCC demonstrated better OS in patients with high CD4+ TILs, no significance was observed in laryngeal or oral cavity SCC [33]. This may suggest that further studies are needed in these anatomical sites to assess the prognostic role of TILs. 

In our sample, we confirmed that the evaluation of TILs provides an advantage in predicting prognosis in patients with OSCC and LUSCC. Despite its high potential, the integration of TILs as a prognostic factor with therapeutic implications remains absent from clinical settings and global oncological treatment guidelines. Moreover, a problem in the standardization of the evaluation technique across different cancer types is slowing the introduction of the evaluation of the tumor immune microenvironment into clinical practice, particularly to better stratify patients’ risk and guide therapeutic decisions. The ease of analyzing TILs may further facilitate the advancement of automated pathological examination. In fact, Pan et al. recently published the validation of an artificial intelligence-driven pathological scoring system for evaluating TILs on H&E-stained whole-slide images of Lung Adenocarcinoma. This system calculates a risk score based on TIL counts in both the cancer epithelium and stroma (WELL score), which demonstrates an independent prognostic value on OS and DFS [34].

Our study confirmed the potential of adopting an Immune-TNM staging approach, akin to the proposal made by Galon et al. in colorectal cancer, although this concept has not yet gained widespread acceptance in clinical oncology disciplines and needs further investigation. However, it is important to acknowledge that to ensure meaningful and comparable results, the adoption of a standardized methodology is imperative for clinical implementation. While TILs hold promise due to their straightforward assessment within H&E staining, CD8+ lymphocytes provide a more precise evaluation of their antitumoral cytotoxic activity. Although this precision was not explicitly demonstrated in our sample, it underscores the potential significance of CD8+ lymphocytes in predicting clinical outcomes.

Our study confirms the prognostic role of the immune system and sheds light on a question previously investigated but not yet realized in a clinical setting. Despite the acknowledged limitations of our study, such as a relatively small sample size and the inclusion of two distinct types of SCC with potentially different underlying biology, our findings highlight the possible variations in the roles of tumor immune macro- and microenvironments in the progression and treatment response of SCC. This may help raise awareness regarding the need for the introduction of immune evaluation in oncological clinical classification.

To think big, we must transcend the boundaries of our current clinical practice, moving beyond guidelines that often compartmentalize various components. Shifting from a concept of tumor cell-autonomous processes to one that embraces systemic host-tumor relationships in malignant diseases requires a broader perspective.

## 5. Conclusions

In conclusion, examining the immune profile of tumors in both the tumor macro- and microenvironment could offer essential and innovative prognostic insights. By generating hypotheses and bringing more attention to this topic, our study emphasizes the potential for incorporating immune evaluation into cancer classification, which could ultimately enhance risk stratification and treatment planning for oncological patients. The results of a worldwide validation of the *Immunoscore* for various cancer types, as previously suggested in the literature, may result in its incorporation as a novel element in cancer classification, leading to the establishment of a new TNM-I (TNM-Immune). 

## Data Availability

The data presented in this study are available in this article and Supplementary Materials.

## Supplementary Materials

The following supporting information can be downloaded at https://www.mdpi.com/article/10.3390/cancers16152759/s1, Table S1: Clinical characteristics of Oral Squamous Cell Carcinoma population; Table S2: Clinical characteristics of Lung Squamous Cell Carcinoma population.
