# Supplementary material for: From Tumor Macroenvironment to Tumor Microenvironment: The Prognostic Role of the Immune System in Oral and Lung Squamous Cell Carcinoma
Source: Cancers (Basel). 2024 Aug 3;16(15):2759. doi: 10.3390/cancers16152759 (PMC11312115; doi:10.3390/cancers16152759)
Supplement: Supplementary file 1 [file cancers-16-02759-s001.zip › cancers-3085504-supplementary.pdf]

Table 1S

Variable	OSCC sample population (n = 104)
Age start treatment (median, range)	67 (19-96)
Male/female (n, %)	51/53 (49%-51%)
Smoke	41 (39.4%)
Alcohol consumption	31 (29.8%)
Eastern Cooperative Oncology Group Performance Status (ECOG PS)	
0	57 (54.8%)
1	18 (17.3%)
2	6 (5.8%)
3	5 (4.8%)
4	3 (2.9%)
Pathological stage	
• I	30 (28.8%)
• II	18 (17.3%)
• III	10 (9.6%)
• IVA	36 (34.6%)
• IVB	2 (1.9%)
• IVC	3 (2.9%)
Treatment	
• Transoral local excision (TLE)	50 (48.1%)
• Compartmental resection w/wo reconstruction	50 (48.1%)
• Total resection w reconstruction	4 (3.8%)
Neck dissection	
• None	23 (22.1%)
• Elective nd	31 (29.8%)
• Sentinel lymph node biopsy (SLNB)	10 (23.8%)
• Therapeutic nd	40 (38.5%)
Adjuvant treatment	
• Radiotherapy	22 (21.2%)
• Chemotherapy	26 (25.0%)

Table 1S: Clinical characteristics of Oral Squamous Cell Carcinoma population

Table 2S

Variable	LUSCC sample population (n = 138)
Age start treatment (median, range)	72 (33-87)
Male/female (n, %)	112/26 (81%-19%)
Smoke	
• Never	7 (5.1%)
• Past	60 (43.5%)
• Current	47 (34.1%)
Comorbidity	
• None	22 (16%)
• Comorbidity artery disease	25 (18%)
• Previous cardiac surgery	5 (4%)
• Current treatment hypertension	49 (36%)
• Current treatment arrhythmia	8 (6%)
• Current treatment cardiac failure	2 (1%)
Pathological stage	
• I	37 (26.7%)
• II	33 (23.9%)
• III	34 (24.6%)

• **IV**	34 (24.6%)
**Surgical procedure**	
• **Bilobectomy**	6 (4.3%)
• **Lobectomy**	48 (34.8%)
• **Pneumonectomy**	17 (12.3%)
• **Segmentectomy**	9 (6.5%)
• **Wedge**	7 (5.1%)
• **Others**	51 (37.0%)
**Lymph node dissection**	
• **None**	62 (44.9%)
• **Radical**	69 (50.0%)
• **Sampling**	7 (5.1%)
• **Other**	0

Table 2S: Clinical characteristics of Lung Squamous Cell Carcinoma population.
